# A chondroblastic osteosarcoma of the coronoid process mimicking a fragmented coronoid process in a dog

**DOI:** 10.1186/s13028-016-0207-6

**Published:** 2016-04-26

**Authors:** Lieve Marie Joseph De Rycke, Wilhelmus Sebastianus Johannes Rasenberg, Koen Cirkel, Henri Jacques Johan van Bree, Ingrid Maria Gielen

**Affiliations:** 1 Department of Veterinary Medical Imaging and Small Animal Orthopaedics, Faculty of Veterinary Medicine, Ghent University, Salisburylane 133, 9820 Merelbeke, Belgium; 2 Veterinair Orthopedisch Centrum Tilburg, Koningsoordlaan 2, 5056 DA Berkel-Enschot, The Netherlands; 3 Pathology Division, Department of Pathobiology, Faculty of Veterinary Medicine, Utrecht University, Yalelaan 1, 3508 TD Utrecht, The Netherlands

**Keywords:** Chondroblastic osteosarcoma, Dog, Ulna, Medial coronoid process, Computed tomography

## Abstract

A 6-year-old Rhodesian Ridgeback was presented with a 1.5 year history of right forelimb lameness. Clinical, radiological and computed tomographic findings suggested the presence of fragmented medial coronoid process. A subtotal coronoidectomy was performed and, due to the atypical appearance of the medial coronoid process on imaging and at surgery, histopathology of the fragments was performed which revealed chondroblastic OS. Ten months after surgery, the dog was re-presented with the same clinical signs and the radiographic changes were suggestive of a recurrence of the OS. Palliative therapy was instigated at the owner’s request. Thirty months after surgery of the neoplasm, the dog was presented with dyspnea. Thoracic radiographs showed lesions consistent with lung metastases. Euthanasia was requested by the owner, who declined post-mortem examination.

## Background

Thoracic limb lameness in young- and middle-aged, large to giant breed, and especially male dogs is most often caused by elbow dysplasia, especially medial coronoid process disease (MCPD) such as fragmented coronoid process (FCP) also named fragmented medial coronoid process (FMCP) or loose coronoid process (LCP) [1, 2]. However, osteosarcoma (OS) of the proximal ulna can cause clinical signs comparable to those of FCP. Ulnar OS is rare in the dog, appears more in males and is affecting respectively 5.81 and 2.35 % of the dogs with skeletal OS [3, 4]. In the latter report, 33 % of the dogs of the proximal third of the ulna was involved. Sivacolundhu et al. [5] examined 30 dogs with ulnar OS and in six dogs (20 %) the proximal third of the ulna was affected. None of these studies provides further information whether the medial coronoid process (MCP) was involved in the neoplastic process.

Clinical signs such as lameness, local swelling of the joint, and pain on manipulation of the elbow can be contributed to several pathologies such as FCP, synovial cell sarcoma or OS of the proximal ulna, but radiographs usually suffice for differentiating these diseases. However, computed tomography (CT) is more sensitive than radiography in detecting medial FCP [1, 6, 7]. In all cases of OS, CT is the technique of preference for evaluating bone destruction and sclerosis [8]. Bone biopsy and/or fine needle aspiration can be performed with or without ultrasound, fluoroscopy or CT guidance and can establish a representative diagnosis in about 90 % of the cases of OS [9–13].

The present study describes the clinical presentation of a dog with chondroblastic OS of the MCP of the elbow, initially diagnosed as a case of FCP. To the authors’ knowledge, this is the first report of a proximal ulnar OS with involvement of the MCP in a dog.

## Case presentation

### Clinical findings

A 6-year-old, female neutered Rhodesian Ridgeback was presented with a 1.5 year history of right forelimb lameness. No previous examinations had been performed. The dog showed a markedly shortened stance phase in its gait at a walk and trot. Significant muscle atrophy of the right shoulder muscles was present. Orthopaedic examination of the right front leg elicited repeatedly a strong pain response on flexion of the shoulder joint with simultaneously digital pressure medial to the greater tubercle in the region of the biceps brachii tendon, while flexing the shoulder the elbow was held in a ‘neutral standing angle’. No joint distention of the right elbow was present and no pain response was found during manipulation of the joint and palpation of radius and ulna. Neurological examination revealed no abnormalities.

### Medical imaging findings

**Fig. 1:**
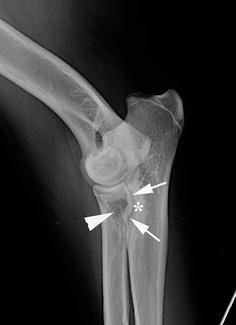
Extended medio-lateral radiographic view of the right elbow. An abnormal shape of the MCP is visible with a more steep to convex delineation (*arrows*). The coronoid process is *blurred* and there is a radiolucent area at the level of the radial head surrounded by a heterogeneous zone (*arrowhead*). The sub-trochlear notch shows sclerosis (*asterisk*). Minor degenerative changes are visible

Radiographs of the shoulders revealed no abnormalities. The extended lateral view of the right elbow showed an abnormally shaped MCP: instead of the normal concave form, a more steep to convex delineation was present. The MCP was blurred and there was a radiolucent area at the level of the radial head surrounded by a heterogeneous zone. The ulnar notch showed sclerosis (Fig. 1).

The dog was referred for diagnostic imaging to the Ghent University Veterinary Faculty, where additional radiographic projections of both elbow joints were made. The previous findings were confirmed and, based on the two radiographic examinations, the presumptive diagnosis of FCP was made.

**Fig. 2:**
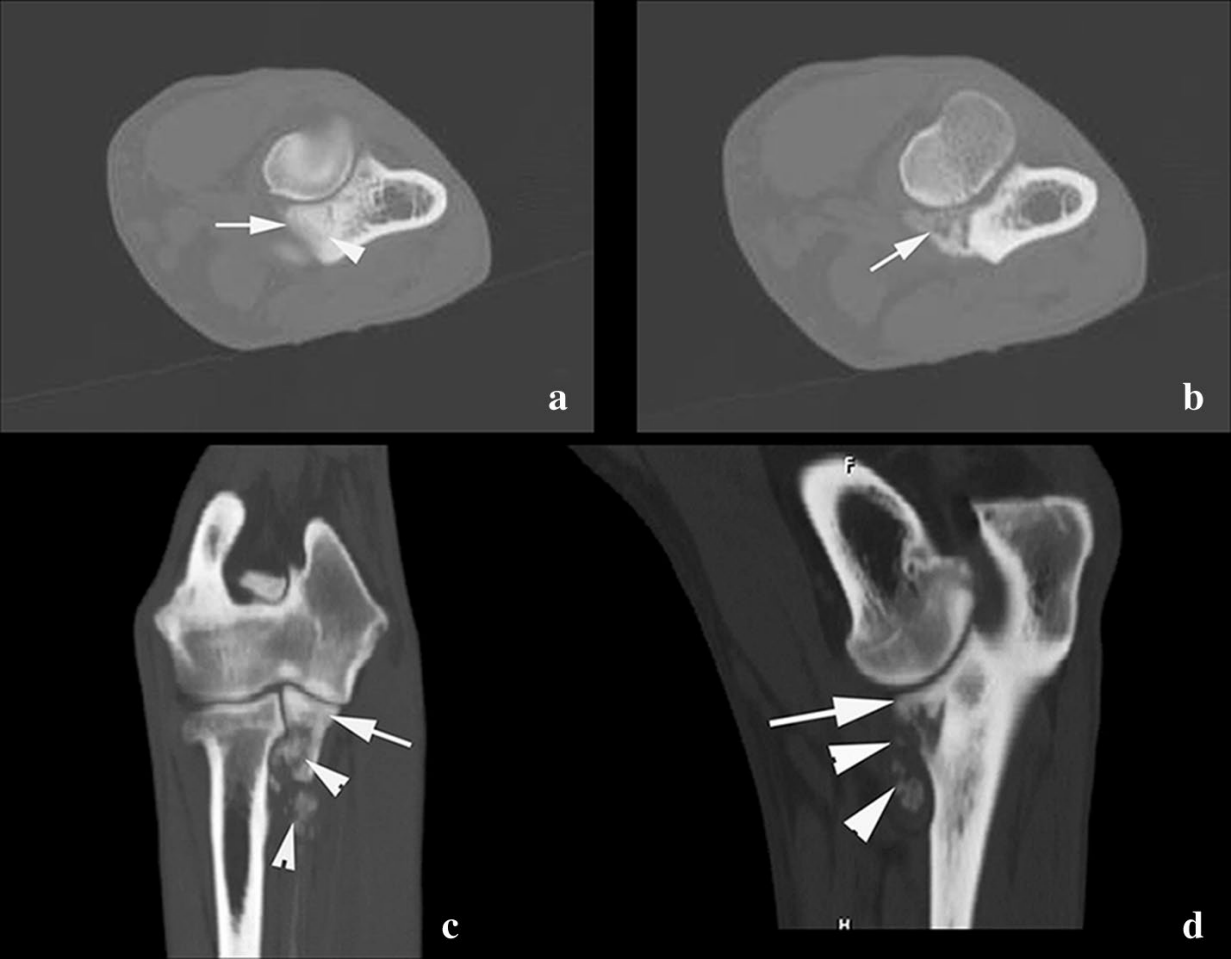
Transverse CT image of the right elbow at the level of the MCP. **a** Abnormal convex delineation of the MCP is present (*arrow*). The sub-trochlear notch shows sclerosis and a small fissure is visible at the level of the MCP (*arrowhead*). More distal transverse CT image (**b**). Demineralization of the MCP is distinct (*arrow*). On the sagittal (**c**) and dorsal (**d**) reformatted images, the surface of the MCP appears to be normal (*arrow*). More distally lysis and heterogeneous aspect of the MCP is noticed (*arrowheads*). Calcified opacities are visible

In addition, CT examination of both elbows was performed. This revealed abnormally steep delineation of the MCP. The sub-trochlear notch showed sclerosis, and a small fissure was visible at the level of the MCP (Fig. 2a). On the more distal transverse CT images (Fig. 2b), demineralization of the MCP associated with several opacities was distinct. These findings were also visible on the sagittal and dorsal reformatted images (Fig. 2c, d).

Clinical and imaging findings suggested the presence of MCP disease, although some atypical findings were present: extensive demineralization associated with several areas with marked new bone formation. Due to these atypical findings, it was decided to conduct a histopathological examination in addition to surgical treatment of the coronoid lesions.

### Surgery

For surgery, a standard medial approach to the elbow joint was used [14]. Macroscopically, only a bone defect at the height of the MCP was noted. The cranial delineation of the MCP was no longer visible. The size of the defect was not measured, but a subtotal coronoidectomy was performed, extending distally 15 mm and caudally 6 mm. The osteotomy surface showed no visible abnormalities. The recovery was uneventful. Meloxicam (Metacam^R^, Boehringer Ingelheim) [0.1 mg/kg once a day per os (po)] was given post-operatively. At 2 weeks post-operatively the lameness substantially decreased. After explanation of the results of pathology at 6 weeks post-operatively the owner declined any follow up examinations and chose to continue the meloxicam as long as this medication could alleviate the pain sufficiently.

### Histopathological findings

**Fig. 3:**
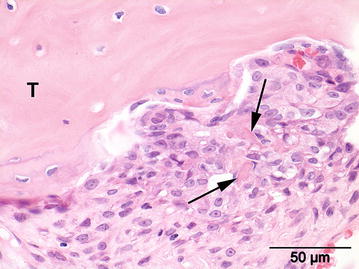
Photomicrograph of the tumor. Moderate pleomorphic population of neoplastic cells revealing small irregular intercellular deposits of hyaline eosinophilic material consistent with osteoid (*arrows*) adjacent to a trabecula of pre-existing bone (*T*). Hematoxylin and eosin staining

Four specimens of the excised bone (15 × 6 mm) were sent for histopathological evaluation. The samples were fixed in 10 % neutral buffered formalin and transferred to a formic acid solution for decalcification. After 3 days of decalcification the tissue samples were routinely processed, paraffin embedded, sectioned at 5 μm and stained with hematoxylin and eosin (HE) for histologic evaluation by a board certified veterinary pathologist. Histopathological evaluation of the tissue samples of the proximal ulna at height of the MCP revealed a non-encapsulated, moderately demarcated, invasive mesenchymal neoplasm, extending through the cortex and through the bony trabeculae into the medulla. The neoplastic cells showed moderate anisocytosis and anisokaryosis, they were spindle-shaped with a small amount of eosinophilic cytoplasm, with distinct cell borders and the cells were arranged in streams. There was one mitotic figure in ten high-power fields (400× magnification). The nuclei were ovoid with a stippled chromatin pattern and contained a single, large, centrally located nucleolus. Multinucleated giant cells were scarce. The neoplastic intercellular matrix was composed of small irregular deposits of hyaline eosinophilic material consistent with osteoid (Fig. 3) that multifocally revealed a moderate amount of mineralization. Also larger areas with cartilaginous matrix were present within the neoplasm.

Based on previously published criteria [15], the neoplasm was diagnosed as a chondroblastic OS. Based on grading criteria defined by Kirpensteijn et al. [16], the dog was classified with low-grade II OS. The edges of the histopathological samples were not free of tumour cells.

### Other information

**Fig. 4:**
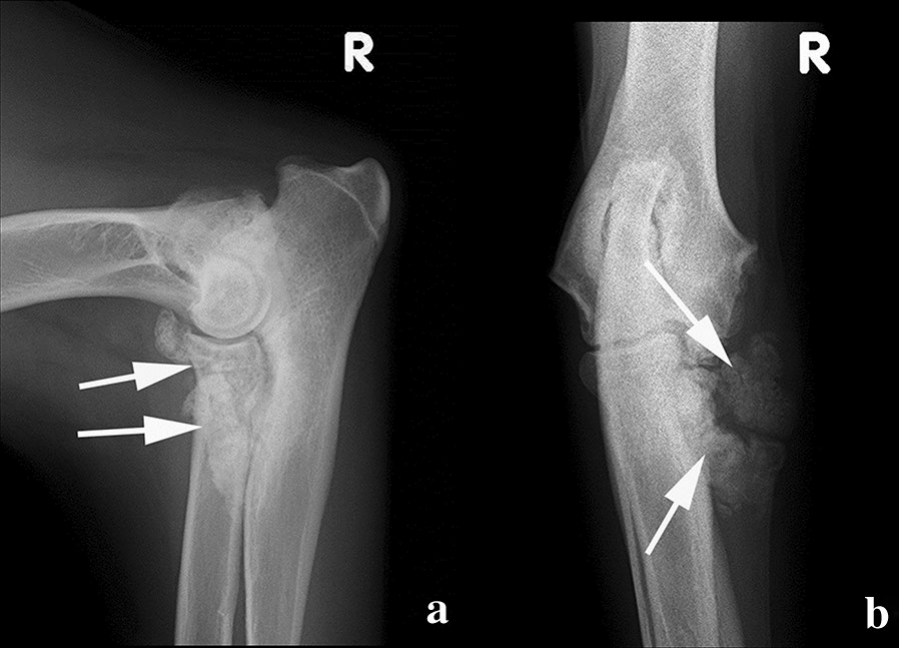
Medio-lateral (**a**) and cranio-caudal (**b**) radiographs of the right elbow 10 months post-operative. Fulminant new bone formation around the area of the medial coronoid is visible (*arrows*)

Several options—including amputation of the right forelimb, chemotherapy or radiation—were suggested to the owner, but were refused. The dog remained slightly lame in the right front leg during 9 months postoperatively despite daily administration of meloxicam. Obviously right forelimb lameness returned after 10 months. As the OS had not been completely excised based on histopathology margins, local recurrence of the tumour was suspected. On the medio-lateral and cranio-caudal radiographs of the right elbow, fulminant new bone formation around the area of the MCP was visible (Fig. 4). This radiographic appearance differed from the expected post-operative images seen after subtotal coronoidectomy, namely a stump coronoid process with little or no bone reaction. The radiographic changes in this case were suggestive of a recurrence of the OS. Biopsy and further therapeutic options were refused by the owner. The dog was treated with daily meloxicam (Metacam^R^, 0.1 mg/kg once a day po) to relieve the clinical signs.

Two and a half years after tumour resection, the dog was presented with severe dyspnea and the right elbow was swollen and painful. Thoracic radiographs showed several large lesions in the lungs suggestive of metastases. The owner requested immediate euthanasia and declined any post-mortem examination.

## Conclusions

Considering the breed of the dog in this case, and based on the localization of the pathology and clinical examination, FCP was suspected initially, as FCP-associated clinical signs are also described in older dogs [17–22].

In addition, radiographic examination led to the assumed diagnosis of FCP in this case, especially due to the lack of aggressive periosteal reaction seen with OS, and the atypical location of OS. However, in contrast to most FCP cases, the joint of this dog was not painful and there was no significant joint distension which is possibly due to the normal surface of the MCP and the absence of osteoarthritis at the time of admission. The CT examination showed extensive demineralization in several areas that normally is not found in FCP. In addition, marked new bone formation was found in the region of the MCP. Therefore, histopathological examination of the removed bone fragment was done. It is possible that the OS in this dog could have developed many months after the origin of the FCP.

The OS of the ulna was classified as a chondroblastic type. In another study, 3.3 % of dogs with ulnar OS were diagnosed with the chondroblastic sub-type [5]. Distinguishing chondroblastic OS from chondrosarcoma (CS) may be difficult based on radiography or CT examination alone, as in this case. Only the histopathological pattern of tumour matrix can provide a useful clue for differentiating chondroblastic OS and CS. The coexistence of bone-forming matrix and a predominating cartilage-forming tumour matrix indicates the presence of chondroblastic OS [23–25]. High-grade CSs cannot be distinguished, either cytomorphologically or histologically, from chondroblastic OS in which tumor osteoid is absent, but can be identified by the absence or sparsity of alkaline phosphatase [24, 25].

The dog in the present case greatly surpassed the median survival time (MST) of 463 days reported in the series of ulna OS cases [5], as the dog survived 930 days after removal of the pathologic fragment. The type of OS (chondroblastic) and low grade of the tumour (low-grade II), might explain the slow progression of clinical signs and metastases. The longstanding lameness before admission and the absence of a painful joint differ from the clinical presentation expected in cases of a high-grade OS of the ulna, which is usually very painful.

The present case report demonstrates the necessity of performing histopathological analysis to distinguish between OS of the MCP and FCP. Based solely on history, clinical signs, radiography and CT, the dog might have been wrongly diagnosed as a case of FCP, despite some atypical radiologic imaging findings. The histopathological examination, however, revealed an OS of the proximal ulna mimicking an FCP.

## Abbreviations

CS: chondrosarcoma
CT: computed tomography
MCPD: medial coronoid process disease
MCP: medial coronoid process
MST: median survival time
OS: osteosarcoma
